# Mirabegron and Anticholinergics in the Treatment of Overactive Bladder Syndrome: A Meta-analysis

**DOI:** 10.1055/s-0043-1770093

**Published:** 2023-07-21

**Authors:** Luisa Gracio Ferreira Sartori, Bruno Monteiro Nunes, Daniela Farah, Leticia Maria de Oliveira, Claudia Cristina Takano Novoa, Marair Gracio Ferreira Sartori, Marcelo Cunio Machado Fonseca

**Affiliations:** 1 Faculdade de Ciências Medicas da Santa Casa de São Paulo, São Paulo, SP, Brazil; 2 Faculdade de Medicina de Marilia, São Paulo, Marília, SP, Brazil; 3 Department of Gynecology, Health Technologies Assessment Center, Universidade Federal de São Paulo, São Paulo, Brazil; 4 Department of Gynecology, Escola Paulista de Medicina, Universidade Federal de São Paulo, São Paulo, SP, Brazil

**Keywords:** antimuscarínicos, metanálise, mirabegrom, bexiga hiperativa, antimuscarinics, meta-analysis, mirabegron, overactive bladder

## Abstract

**Objective** To compare the use of mirabegron with anticholinergics drugs for the treatment of overactive bladder (OB).

**Data Source** Systematic searches were conducted in EMBASE, PUBMED, Cochrane, and LILACS databases from inception to September 2021. We included RCTs, women with clinically proven OB symptoms, studies that compared mirabegron to antimuscarinic drugs, and that evaluated the efficacy, safety or adherence.

**Data Collection** RevMan 5.4 was used to combine results across studies. We derived risk ratios (RRs) and mean differences with 95% CIs using a random-effects meta-analytic model. Cochrane Collaboration Tool and GRADE was applied for risk of bias and quality of the evidence.

**Data Synthesis** We included 14 studies with a total of 10,774 patients. Fewer total adverse events was reported in mirabegron group than in antimuscarinics group [RR 0.93 (0.89–0.98)]. The risk of gastrointestinal tract disorders and dry mouth were lower with mirabegron [RR 0,58 (0.48–0.68); 9375 patients; RR 0.44 (0.35–0.56), 9375 patients, respectively]. No difference was reported between mirabegron and antimuscarinics drugs for efficacy. The adherence to treatment was 87.7% in both groups [RR 0.99 (0.98–1.00)].

**Conclusion** Mirabegron and antimuscarinics have comparable efficacy and adherence rates; however, mirabegron showed fewer total and isolated adverse events.

## Resumo

**Objetivo** Comparar o uso de mirabegrom com anticolinérgicos para o tratamento da bexiga hiperativa (BH).

**Fonte de Dados** Buscas sistemáticas foram realizadas nas bases de dados EMBASE, PUBMED, Cochrane e LILACS desde o início até setembro de 2021. Incluímos ECR, mulheres com sintomas de BH clinicamente comprovados, estudos que compararam mirabegrom a medicamentos antimuscarínicos e avaliaram a eficácia, segurança ou adesão.

**Coleta de Dados** RevMan 5.4 foi usado para combinar os resultados entre os estudos. Derivamos razões de risco (RRs) e diferenças médias com intervalo de confiança (IC) de 95% usando um modelo meta-analítico de efeitos aleatórios. Cochrane Collaboration Tool e GRADE foi aplicado para risco de viés e qualidade da evidência.

**Síntese dos Dados** Foram incluídos 14 estudos com um total de 10.774 pacientes. Menos eventos adversos totais foram relatados no grupo mirabegrom do que no grupo antimuscarínicos [RR: 0,93 (0,89–0,98)]. O risco de distúrbios do trato gastrointestinal e boca seca foram menores com mirabegrom [RR: 0,58 (0,48–0,68); 9.375 pacientes; RR: 0,44 (0,35–0,56), 9.375 pacientes, respectivamente]. Nenhuma diferença foi relatada entre mirabegrom e drogas antimuscarínicos para eficácia. A adesão ao tratamento foi de 87,7% em ambos os grupos [RR: 0,99 (0,98–1,00)].

**Conclusão** Mirabegrom e antimuscarínicos têm eficácia e taxas de adesão comparáveis, porém o mirabegrom apresentou menos eventos adversos totais e isolados.

## Introduction

Overactive bladder is a syndrome characterized by urinary urgency, increased daytime urinary frequency, nocturia, with or without urge urinary incontinence, and no evidence of infection or other proven diseases. 1 It is a condition that impairs patients' quality of life by causing depression, anxiety, frustration, low self-esteem, and social isolation. 2

The economic impact of overactive bladder is exceptionally high, taking into account both the diagnosis and treatment of the disease, as well as the consequences of the disease, and it tends to increase due to longer life expectancy, as overactive bladder has a higher prevalence with increasing age. 3 4

Overactive bladder treatment can be pharmacological or non-pharmacological. Behavioral and educational treatments are examples of non-pharmacological treatments. Behavioral and educational interventions, such as limiting the intake of bladder irritants like caffeine and alcohol, bladder training, and urgency suppression techniques like pelvic floor muscle exercises, should be included in the therapeutic plan and offered at the outset. These interventions can be used in conjunction with pharmacological treatment, effective in most overactive bladder patients and are the first line of treatment. 5 In case of treatment failure, another possibility would be botulinic toxin or neuromodulation. 5

Some drugs are both safe and effective in the treatment of overactive bladder. Anticholinergic drugs increase bladder capacity by lowering detrusor muscle tone during the filling phase. 6 Among the anticholinergic drugs mentioned are oxybutynin, tolterodine, darifenacin, and solifenacin. Anticholinergic drugs block and inhibit muscarinic receptors located not only on the bladder. That may lead to adverse events such as dry mouth and constipation and lead to loss of follow up. Anticholinergic drugs can also penetrate the blood-brain barrier and affect cognition, especially in the elderly.

Another class of drugs used to treat overactive bladder is β-3-adrenergic agonists, which directly inhibit afferent nerve activation through the activation of β type adrenergic receptors at the bladder, causing vesical relaxation. This class of drug has less adverse effects on the salivary glands, therefore, less symptoms such as dry mouth or cognition dysfunction. Mirabegron is a β-3-adrenergic drug that is an alternative to anticholinergic medications. 7

There is disagreement about the efficacy, tolerability, safety, and adherence to anticholinergic drugs and mirabegron treatment. As a result, comparing these classes of medications in the treatment of overactive bladder is critical.

The goal of this study was to conduct a systematic review followed by a meta-analysis of the efficacy, safety, and adherence to treatment with β-3-adrenergic drugs versus anticholinergics for overactive bladder in women, using evidence from randomized controlled clinical trials.

## Methods

The systematic review and meta-analysis were performed following the Preferred Reporting Items for Systematic Reviews and Meta-Analyses (PRISMA) recommendations. PROSPERO was used to register the study protocol (CRD42019135355).

The EMBASE, Medline (PUBMED), Cochrane, and LILACS databases were used to find the studies. The search included all articles published from inception until October 2022. “Overactive Bladder,” “Overactive Detrusor Function,” “Overactive Urinary Bladder,” “Overactive Detrusor,” “Mirabegron,” “Oxybutynin,” “Solifenacin,” “Tolterodine,” “Darifenacin” were the keywords used in the search strategy. The full search strategy can be found in supplementary material (SM) 1.

Two different reviewers analyzed the articles found by the search strategy based on the title and abstract, and the publications that met the eligibility criteria were then thoroughly reviewed. The included studies met the following criteria: (1) randomized controlled trial, (2) study with women over the age of 18 with clinically proven overactive bladder symptoms, (3) mirabegron versus antimuscarinics comparative studies (oxybutynin, darifenacin, tolterodine, or solifenacin). This review only included complete published studies in either Spanish, Italian, French, Portuguese or English language.

We did not include articles that only evaluated men or children, pregnant women, or did not assess the selected outcomes. There was no set minimum or maximum follow-up time for this meta-analysis. We excluded duplicate publications, conference proceedings abstracts, case studies, letters to the editor, review articles, editorials, comments, and animal studies. The outcomes of interest were efficacy, safety, and adherence. The drugs' efficacy was determined by the reduction in the number of urinations, episodes of urge incontinence, urgency, and incontinence within 24 hours (unspecified urinary losses). The total number of adverse events reported by patients and the most relevant adverse events assessed separately, such as gastrointestinal tract disorders (constipation, diarrhea, dyspepsia, nausea, dry mouth), nervous system disorders (headache, dizziness, drowsiness, blurred vision), and others (nasopharyngitis, urinary retention, urinary tract infection, cardiac conditions, hypertension), evaluated the safety outcome. Cardiac disorders included atrial fibrillation, tachycardia, palpitation, supraventricular extrasystole, atrial flutter, extrasystole, QT interval prolongation, arrhythmias, tachyarrhythmia, left bundle branch block, sinus arrhythmia, and cardiac arrhythmia. Based on the number of patients who reported them, all adverse events were expressed as a percentage of the total number of patients enrolled in the study.

The adherence outcome was calculated by dividing the total number of patients who completed the study by the total number of patients who participated in the study. The risk of bias of individual studies was assessed using the Cochrane Collaboration Tool (RoB-1) for RCTs and the certainty of the evidence was assessed by the Grading of Recommendations, Assessment, Development, and Evaluations (GRADE).

Reviewers extracted data from the studies using a standardized form using Excel®. The Cochrane Collaboration Review Manager statistical software (Review Manager version 5.4.1) combined results from the various studies and applied the random effects meta-analytic model using the inverse variance method in all calculations. The outcomes of categorical variables were expressed as a risk ratio (RR) and the outcomes of continuous variables as a mean difference (MD), both with 95% confidence intervals (CI). The Q test (X 2 test) and the I ^2^ test were used to assess heterogeneity. Subgroup analysis was performed by mirabegron dosage, 25 mg and 50 mg. Publication bias was assessed using Begg's funnel plot in RevMan version 5.4.1, where each OR point estimate was plotted against its corresponding standard errors (SE) on a logarithmic scale.

## Results

**Fig. 1:**
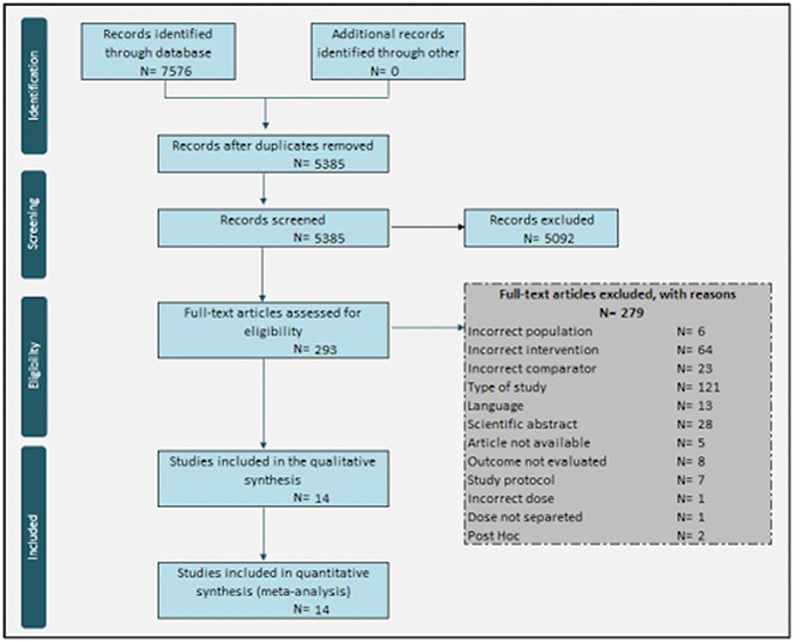
PRISMA Flowchart.

Initially, 7,698 studies were found, with 2,207 of them being duplicates, leaving 5,491 articles to be assessed by titles and abstracts. After reviewing, there were 293 studies to be evaluated for a full-text review. This meta-analysis included 14 studies with a total of 10,774 patients ( Fig. 1 ). 8 9 10 11 12 13 14 15 16 17 18 19 20 21 Chart 1 describes the main characteristics of the included studies. A total of 6 studies with 5,535 patients, compared mirabegron (25–50mg) to solifenacin (2.5mg-5mg-10mg) 12 13 16 17 19 21 and 8 studies with 5,239 patients, compared mirabegron (25–50mg) to tolterodine ER 4mg. 8 9 10 11 14 15 18 20 There were no studies that compared mirabegron to oxybutynin or darifenacin.

**Chart 1:** Essential characteristics of the included studies

Author, year	Country	Intervention group	Comparator group	Number of patients in the intervention group		Number of patients in the comparator group	Percentage of women in the intervention group		Percentage of women in the comparator group	Age of patients in the intervention group (years), mean (SD)		Age of patients in the comparator group (years), mean (SD)
				MIRA 25mg	MIRA 50mg		MIRA 25mg	MIRA 50mg		MIRA 25mg	MIRA 50mg	
Chapple et al. (2013) 8	Multicentric	MIRA 25 e 50mg	TOL ER 4mg	167	167	85	88%	89.2%	81.2%	57,2 (12,1)	56,9 (12,5)	56,6 (12,8)
Chapple et al. (2013) 9	Multicentric	MIRA 50mg	TOL ER 4mg	NA	812	812	NA	74.1%	73.9%	NA	59.2 (12.6)	59.6 (12.5)
Khullar et al. (2013) 10	Multicentric	MIRA 50mg	TOL ER 4mg	NA	493	495	NA	72.4%	72.9%	NA	59.1 (12.3)	59.1 (12.9)
Yamaguchi et al. (2014) 11	Multicentric	MIRA 50mg	TOL ER 4mg	NA	369	368	NA	84.3%	82.6%	NA	58.3 (13.9)	58.3 (13.7)
Abrams et al. (2015) 12	Multicentric	MIRA 25 e 50mg	SOL 2.5mg, 5mg e 10mg	77	78	313	67.5%	66.67%	66.1%	55.2 (14.5)	53.4 (14.0)	54,88 (13,9)
Batista et al. (2015) 13	Multicentric	MIRA 50mg	SOL 5mg	NA	936	934	NA	76.1%	75.9%	NA	56.7 (14.3)	57.4 (13.6)
Kuo et al. (2015) 14	Multicentric	MIRA 50mg	TOL ER 4mg	NA	338	333	NA	67.5%	64%	NA	54.3 (14.2)	53.9 (14.5)
Kuo et al. (2015) 15	Multicentric	MIRA 50mg	TOL ER 4mg	NA	76	74	NA	61.8%	59.5%	NA	59.0 (15.1)	56.4 (15.8)
Vecchioli Scaldazza and Morosetti (2016) 16	Italy	MIRA 50mg	SOL 5mg	NA	31	29	NA	100%	100%	NA	56 (6.25)	58 (7.0)
Herschorn et al. (2017) 17	Multicentric	MIRA 25 e 50mg	SOL 5mg	423	422	423	77.3%	76.5%	78.3%	56.9 (13.6)	56.7 (13.3)	58.2 (12.8)
Hsiao et al. (2018) 18	Multicentric	MIRA 50mg	TOL ER 4mg	NA	12	12	NA	100%	100%	NA	53 (3.5)	48,75 (2.6)
Gratzke et al. (2018) 19	Multicentric	MIRA 50mg	SOL 5mg	NA	302	299	NA	79%	81%	NA	61 (10.7)	60 (11.2)
Staskin et al. (2018) 20	Multicentric	MIRA 25mg	TOL ER 4mg	316	NA	310	73.4%	NA	75.2%	53.4 (13.9)	NA	53.2 (13.7)
White et al. (2018) 21	Multicentric	MIRA 25 e 50mg	SOL 5mg	423	422	423	77,3%	76,5%	78,3%	56,9	56,7	58,2

Abbreviations: MIRA, Mirabegron; NA, Not available; SOL, Solifenacin; SD, Standard deviation; TOL, Tolterodine.

According to the Cochrane collaboration tool (RoB-1), the overall risk of bias of the included studies was low. Majority of the studies had a high risk of bias for “other sources of bias.” The GRADE considered most outcomes evaluated to be low quality of evidence. There was no asymmetry in Begg's funnel-plot for any outcome evaluated, therefore no publication bias was found. There were no differences between the use of mirabegron compared with antimuscarinics concerning efficacy outcomes ( Table 1 ).

**Table 1:** Results according to the outcomes of interest

		Number of included studies	Number of patients evaluated		Estimated Effect (95% CI)	GRADE	Heterogeneity	
			Mirabegron	Antimuscarinics			I ^2^	P
Efficacy	Number of urinations/24h	9	4232	3960	MD: -0.00 (-0.16 to 0.16)	Low	47%	0.04
	Urge incontinence episodes/24h	7	2281	1906	MD: 0.08 (-0.02 to 0.17)	Low	0%	0.95
	Emergency episodes/24h	8	3926	3656	MD: 0.04 (-0.10 to 0.19)	Low	0%	0.55
	Episodes of urine leakage/24h	9	2808	2477	MD: 0.06 (-0.09 to 0.20)	Low	34%	0.13
Safety	Total Side Effects	11	1879/4979 (37.7%)	1898/4476 (42.4%)	RR: 0.93 (0.89 to 0.98)	Low	7%	0.38
	Gastrointestinal tract disorders (general)	10	340/4939 (6.9%)	556/4436 (12.5%)	RR: 0.58 (0.48 to 0.68)	Low	31%	0.14
	Dry mouth	10	180/4939 (3.6%)	400/4436 (9.0%)	RR: 0.44 (0.35 to 0.56)	Low	34%	0.11
	Constipation	10	120/4939 (2.4%)	119/4436 (2.7%)	RR: 0.95 (0.73 to 1.22)	Low	0%	0.97
	Diarrhea	2	18/1117 (1.6%)	20/1115 (1.8%)	RR: 0.90 (0.48 to 1.69)	Low	0%	0.78
	Dyspepsia	4	10/2265 (0.4%)	14/1755 (0.8%)	RR: 0.74 (0.32 to 1.72)	Very Low	0%	0.97
	Nausea	2	10/648 (1.5%)	9/410 (2.2%)	RR: 0.76 (0.30 to 1.89)	Low	0%	0.91
	CNS disorders (general)	9	217/4560 (4.8%)	172/4061 (4.2%)	RR: 1,14 (0.93 to 1.39)	Low	0%	0.81
	Headache	7	117/3349 (3.5%)	93/3267 (2.8%)	RR: 1.22 (0.93 to 1.61)	Low	0%	0.91
	Dizziness	5	38/1967 (1.9%)	34/1884 (1.8%)	RR: 1.00 (0.61 to 1.62)	Low	0%	0.55
	Somnolence	3	44/1469 (3.0%)	30/1051 (2.9%)	RR: 1.03 (0.58 to 1.82)	Low	28%	0.25
	Visual blur	4	20/2255 (0.9%)	18/1995 (0.9%)	RR: 1.12 (0.60 to 2.08)	Low	0%	0.69
	Nasopharyngitis	5	81/2131 (3.8%)	76/2292 (3.3%)	RR: 1.18 (0.87 to 1.61)	Low	0%	0.96
	Urinary retention	5	4/3217 (0.1%)	10/2797 (0.4%)	RR: 0.41 (0.14 to 1.26)	Low	0%	0.77
	Urinary tract infection	7	165/3795 (4.3%)	183/3605 (5.1%)	RR: 0.87 (0.70 to 1.07)	Low	0%	0.97
	Cardiac disorders	10	147/4939 (3.0%)	142/4436 (3.2%)	RR: 1.00 (0.74 to 1.35)	Low	18%	0.26
	Hypertension	9	175/4610 (3.8%)	202/4351 (4.6%)	RR: 0.93 (0.76 to 1.13)	Low	0%	0.50
Adherence		11	4175/4762 (87.7%)	3549/4046 (87.7%)	RR: 0.99 (0.98 to 1.00)	Low	2%	0.43

Abbreviations: CI, Confidence interval; CNS, Central nervous system; MD, Mean difference; RR, Risk ratio.

**Fig. 2:**
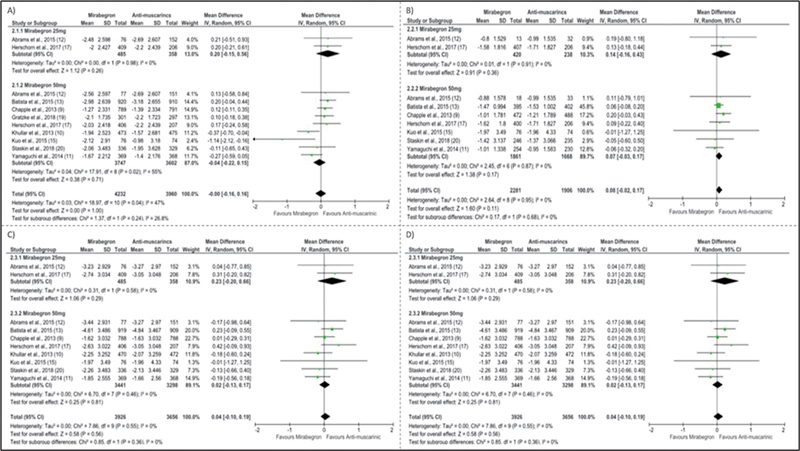
Forest plots showing efficacy outcome: ( **A** ) Number of urinations every 24h, ( **B** ) Urge incontinence episodes, ( **C** ) Urgency; D: Urinary leakage episodes.

Nine studies evaluated the decrease in the number of urinations every 24 hours which included 8,192 patients [MD: -0.00 (-0.16 to 0.16); I ^2^ = 47%] ( Fig. 2A ). 9 10 11 12 13 15 17 19 20 Seven studies with 4,187 patients evaluated the reduction in urge incontinence episodes [MD: 0.08 (-0.02 to 0.17); I ^2^ = 0%] ( Fig. 2B ). 9 11 12 13 15 17 20 Eight studies with a total of 7,582 patients assessed the urgency outcome [MD: 0.04 (-0.10 to 0.19); I ^2^ = 0%] ( Fig. 2C ). 9 10 11 12 13 15 17 20 Nine studies analyzed 5,285 patients with unspecified urinary losses [MD: 0.04 (-0.10 to 0.19); I ^2^ = 0%] ( Fig. 2D ). 9 10 11 12 13 15 17 19 20

**Fig. 3:**
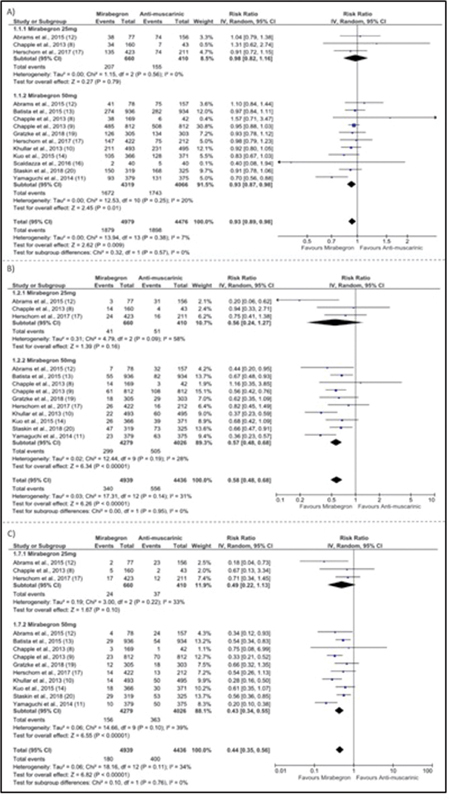
Forrest plots showing safety outcomes. ( **A** ) Total number of side effects; ( **B** ) Gastrointestinal side effects; ( **C** ) Presence of dry mouth.

Corrigir as citações dos autores no interior da Figura 2 como indicado abaixo:

Eleven studies looked at drug side effects. 8 9 10 11 12 13 14 16 17 19 20 In terms of total adverse events, patients treated with mirabegron had fewer adverse events than patients treated with antimuscarinics [RR: 0.93 (0.89–0.98); I ^2^ = 7%; 9,455 patients] ( Fig. 3A ). Ten studies with 9,375 patients, examined the number of patients with gastrointestinal tract disorders which was lower with the use of mirabegron [RR: 0.58 (0.48–0.68); I ^2^ = 31%] ( Fig. 3B ). 8 9 10 11 12 13 14 17 19 20 Patients taking mirabegron had a lower incidence of dry mouth [RR: 0.44 (0.35–0.56); I ^2^ = 34%; 9,375 patients] ( Fig. 3C ). However, when the reviewers examined only the studies with the 25mg dose of mirabegron, these differences were not found ( Table 1 ).

**Fig. 4:**
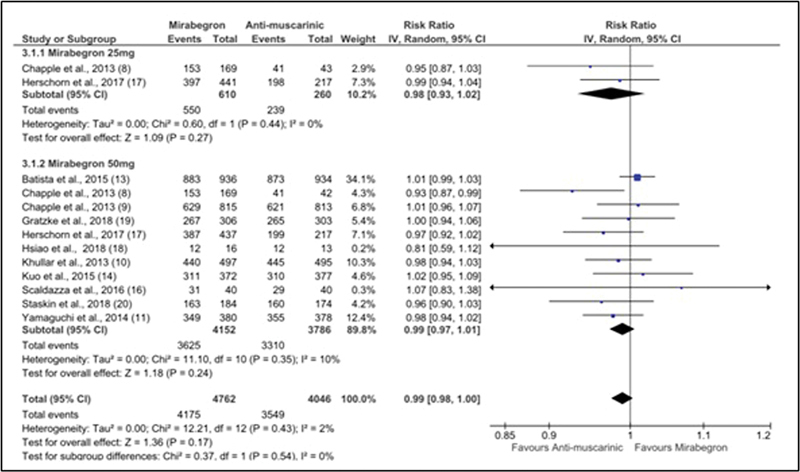
Forest plot showing adherence outcome.

Corrigir as citações dos autores no interior da Figura 3 como indicado abaixo:

When the reviewers compared other adverse events such as constipation, diarrhea, dyspepsia, and nausea separately, there was no difference between the two drugs evaluated ( Table 1 ). There were no differences in adverse events on the central nervous system or elsewhere between patients taking mirabegron or anticholinergics ( Table 1 ). Eleven articles involving 8,808 patients investigated patient's treatment adherence. 8 9 10 11 12 13 14 16 17 18 19 20 There was no difference in the treatment of overactive bladder between patients who used mirabegron and those who used antimuscarinics [RR: 0.99 (0.98–1.00); I ^2^ = 2%] ( Fig. 4 ). A total of 87.7% of the patients in both groups completed the proposed treatments.

Corrigir as citações dos autores no interior da Figura 4 como indicado abaixo:

## Discussion

Overactive bladder syndrome is a clinical condition that decreases the quality of life. 2 After behavioral or physical therapy measures, drug treatment is considered the second line of treatment. Anticholinergics and, more recently, β-3-adrenergic drugs are the most commonly used classes of drugs in the treatment of overactive bladder. 5 Adherence to drug treatment, on the other hand, can be reduced due to side effects, ineffectiveness, or even the cost of medications. 22 23

Anticholinergic treatment is estimated to be discontinued in 4% to 31% of patients after 12 weeks, primarily due to adverse events. Its effect on dry mouth and constipation as a cause of treatment discontinuation is well documented. 7 24

Other anticholinergics, such as solifenacin, darifenacin, and tolterodine, have been developed to reduce side effects while improving adherence and therapeutic outcomes. 5 One of the most commonly used drugs in treating overactive bladder in Brazil is oxybutynin, which has satisfactory efficacy results but has adverse events limiting its use, especially in older people, particularly compromising cognition. 25 In this review, no difference was found between mirabegron and anticholinergic drugs when it comes to adverse effects in the central nervous system, such as cognition dysfunction. These symptoms are more frequent in older patients in chronic use of anticholinergics. In this present review, the average follow-up of the studies was 14 weeks, which might be insufficient to analyze cognitive impairment.

Mirabegron, a β-3 adrenergic receptor agonist, was recently approved to treat overactive bladder, making it an alternative to anticholinergic drugs. This medication acts directly on the detrusor muscle by activating β-3 adrenergic receptors and indirectly on the parasympathetic nerves. 26

There is some debate over which of these would be the best class of drugs to use in the treatment of overactive bladder. As a result, the goal of this meta-analysis was to determine which class of medication is more effective, safe, and has the highest adherence for the treatment of women with overactive bladder.

This meta-analysis demonstrated that mirabegron causes less dry mouth than antimuscarinics, as several authors observed. 25 27 28

Furthermore, because anticholinergics cross the blood-brain barrier, their use in elderly patients should be cautious. On the other hand, mirabegron is safer in terms of central nervous system side effects. 29 However, this meta-analysis showed no difference between adverse events on the central nervous system, such as dizziness, headache, and somnolence, the only ones described in the studies. This finding could be due to a failure to adequately assess cognitive symptoms, as well as, a failure to differentiate adverse events by age group in the studies examined.

Although the meta-analysis discovered that mirabegron and antimuscarinics have comparable efficacy and adherence rates, mirabegron was safer in terms of total side effects as well as side effects such as general gastrointestinal disorders and dry mouth.

This systematic review's strength was that it only included randomized studies that compared mirabegron to other anticholinergics. The main flaw was that the reviewers found no studies that only looked at women. Studies that did not include adult women were excluded from this meta-analysis to reduce this bias. However, studies that included men did not present outcomes based on patient gender, nor did they report gender inclusion percentages. Furthermore, the articles included in this meta-analysis were deemed low quality because some of the authors had commercial ties to pharmaceutical companies that produced the analyzed drugs. This is a crucial bias to consider, in our opinion. Adherence outcome also has important limitation in the interpretation due to different treatment duration between studies, which ranged from 12 weeks to 18 weeks.

A recent meta-analysis reported that treatment adherence rates vary depending on age, gender, medication type, and the study's year. 30 We believe that studies should be conducted based on the gender of the patients because the causes of overactive bladder and therapeutic responses may differ depending on the patient's hormonal state.

No articles compared mirabegron to oxybutynin, one of the most commonly used drugs in Brazil to treat overactive bladder. However, several studies show that solifenacin and tolterodine outperform oxybutynin, particularly in terms of adverse events. 31 32 33 As a result, it is reasonable to believe that mirabegron beats oxybutynin in this regard.

## Conclusion

As a result, the current meta-analysis observed that while mirabegron and antimuscarinics have comparable efficacy and adherence rates, mirabegron has fewer total and isolated side effects to treat women with overactive bladder. Studies focusing solely on women could shed lighter in the efficacy, safety, and adherence of medications in this population.
